# The Optimization of Alternating Current Electrospun PA 6 Solutions Using a Visual Analysis System

**DOI:** 10.3390/polym13132098

**Published:** 2021-06-25

**Authors:** Tomas Kalous, Pavel Holec, Jakub Erben, Martin Bilek, Ondrej Batka, Pavel Pokorny, Jiri Chaloupek, Jiri Chvojka

**Affiliations:** 1 Department of Nonwovens and Nanofibrous Materials, Faculty of Textile, Technical University of Liberec, 461 17 Liberec, Czech Republic; pavel.holec@tul.cz (P.H.); jakub.erben@tul.cz (J.E.); pavel.pokorny@tul.cz (P.P.); jiri.chaloupek@tul.cz (J.C.); jiri.chvojka@tul.cz (J.C.); 2 Department of Textile Machine Design, Faculty of Mechanical Engineering, Technical University of Liberec, 461 17 Liberec, Czech Republic; martin.bilek@tul.cz

**Keywords:** nanofibers, AC electrospinning, relaxation time, productivity, polymer solution

## Abstract

The electrospinning process that produces fine nanofibrous materials have a major disadvantage in the area of productivity. However, alternating current (AC) electrospinning might help to solve the problem via the modification of high voltage signal. The aforementioned productivity aspect can be observed via a camera system that focuses on the jet creation area and that measures the average lifespan. The paper describes the optimization of polyamide 6 (PA 6) solutions and demonstrates the change in the behavior of the process following the addition of a minor dose of oxoacid. This addition served to convert the previously unspinnable (using AC) solution to a high-quality electrospinning solution. The visual analysis of the AC electrospinning of polymeric solutions using a high-speed camera and a programmable power source was chosen as the method for the evaluation of the quality of the process. The solutions were exposed to high voltage applying two types of AC signal, i.e., the sine wave and the step change. All the recordings presented in the paper contained two sets of data: firstly, camera recordings that showed the visual expression of electrospinning and, secondly, signal recordings that provided information on the data position in the signal function.

## 1. Introduction

The current high demand for ultra-fine fibers has led to the rapid development of several technological approaches for the production of such materials. The dominant approach to the production of nanofibers currently comprises electrospinning technology that, in the majority of cases, involves a direct current (DC) power source [1]. DC electrospinning can be divided into two groups, i.e., needle electrospinning [2] and free surface (needleless) electrospinning [3]. The further development of the latter subsequently led to the introduction of Nanospider technology [4], while the former enabled the small scale experiments. Initially, the single-fluid systems were electro-spun [1] and, to date, are the most used method [5]. Development of complex forms of DC electrospinning of solutions contained techniques such as side-by-side [6], coaxial [7], tri-axial [8,9], or multiple-fluid systems spinning [10,11]. However, such rapid development in the spinning systems was not followed by a detailed examination of the spinning processes. This paper offers a simple visual method of single fluid process observation that could also be useful for the more complex methods mentioned above.

In general, DC electrospinning uses a charged electrode covered with a polymeric solution and an electrically active collector (grounded or powered by an opposite charge) for the production of nanofibers. The solution is charged with a high voltage, which leads to the destabilization of the liquid surface, the creation of Taylor cones, and the subsequent formation of stable jets [12]. The necessity for an electrically active collector in DC technology is driven by the attraction of the electro-spun fibers to dissipate their accumulated charge, and the advantage of this phenomenon comprises the potential to direct the nanofibrous product to a certain area. This means that all the materials produced via DC technology consist of thin layers of densely deposited nanofibers.

The subsequent development of DC electrospinning uncovered several areas for further exploration, one of which concerned the so-called “relaxation time” [12,13], a phenomenon that relates to the requirement of polymeric solutions for a certain amount of time ranging from milliseconds to, in extreme cases, minutes prior to the beginning of the spinning process. This time interval occurs between the initial application of the voltage and the creation of one or multiple jets, being caused by the destabilization of the surface of the liquid polymer as mentioned above. Initially, the offset time of the electrospinning process was described using a DC power source [14,15]. However, it was subsequently assumed that using an AC signal at a suitable frequency would be sufficient from the time and energy viewpoints to destabilize the liquid surface and thus initiate the offset of the electrospinning process. This has been demonstrated in other studies [16,17] that revealed the potential of AC electrospinning with an electrically active collector. Another study [18] described a possibility to change the shape of the signal and frequency for obtaining a material with better drug release potential. Further research on the relaxation time revealed that AC electrospinning does not require an electrically active collector, which led to the development of the collector-less electrospinning process [19]. The materials produced via this technology exhibit different properties to those produced via the classic DC variant, i.e., they are bulky and can be used to cover any type of material, even without a charge or grounding. This is due to the principle of AC spinning, which serves to alternate the emission of positive and negative fibers, thus leading to the self-dissipation of the total charge in the products. The resulting electrically-neutral product is transferred away from the electrode via the ionic wind [20,21]. Due to their bulkiness, fibers electro-spun by means of AC technology can be used for filtration [22,23], tissue engineering purposes [24,25], or as one of the components of composites [26] that are in some cases spinnable with the high dose of particles [27] without reduction of production rates.

To date, no research paper has described the correlation between the type of the AC signal and the visual manifestation of the electrospinning process. This area of research is potentially interesting in terms of enhancing the productivity of AC electrospinning. This can be achieved by determining the optimal shape or frequency of the AC signal or via the modification of the electro-spun solution, as described in our previous study [28]. This paper thus addresses and presents a number of potential approaches to this issue.

## 2. Materials and Methods

### 2.1. Materials

All the experiments employed Polyamide 6 (PA 6) Ultramid B27 (M_w_ 66.360 g/mol) provided by BASF company. The solvent system consisted of 98% of formic acid (Penta Chemicals, Czech Republic) and 99% of acetic acid (Penta Chemicals, Prague, Czech Republic) mixed at a wt% ratio of 1:1. The additives that led to the enhancement of the electrospinning process consisted of two oxoacids, i.e., 96% of sulfuric acid (Penta) and 99% of methane-sulfonic acid (Sigma Aldrich, Hamburg, Germany).

### 2.2. Preparation of Solutions

The basic solution used for all the experiments, was 10% (wt%) PA 6, that was dissolved in the formic and acetic acids (1:1 wt%). Initially, a set of calibration experiments was performed so as to determine the doses of the additives. With respect to the detailed observation and for further measurement purposes, two oxoacids were chosen from the set of experiments on the basis of their performance in terms of significantly enhancing the electrospinning process, i.e., sulfuric (H_2_SO_4_) and methane-sulfonic acids (CH_3_SO_3_H). The acids were added in small doses to the basic solution. The dose of H_2_SO_4_ was 0.2 mol/L, and that of CH_3_SO_3_H was 0.37 mol/L [28]. These values were found to result in the optimal AC electrospinning process.

### 2.3. Electrode System and High Voltage

Aimed at maintaining standard electrospinning experimental conditions, the dosing of the polymer was ensured via a custom-made screw pump [26] that allowed for the continual overflow of the polymeric solution and the creation of a film over the whole surface of the electrode. The signal was generated using an Owon AG 1022 function generator and was transformed to high voltage using a TREK 50/12 high-voltage amplifier. The high-voltage value was set at 42 kV (amplitude) with a frequency of 50 Hz. The voltage amplitude was set at 42 kV due to the necessity to exceed the critical voltage at the start of the spinning process. The amount of energy provided to the system with the sinus wave was not equal to the energy provided by the step function. However, if the effective values of each voltage function had been the same, we would probably have been faced with a problem in terms of traversing the critical voltage.

### 2.4. High-Speed Camera and Light

**Figure 1:**
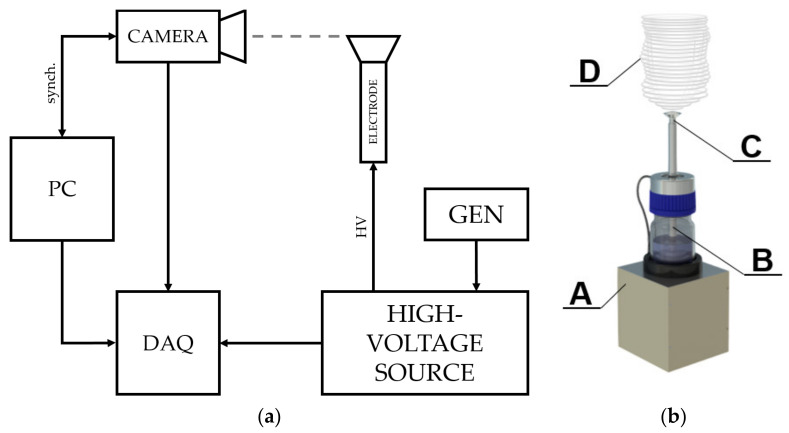

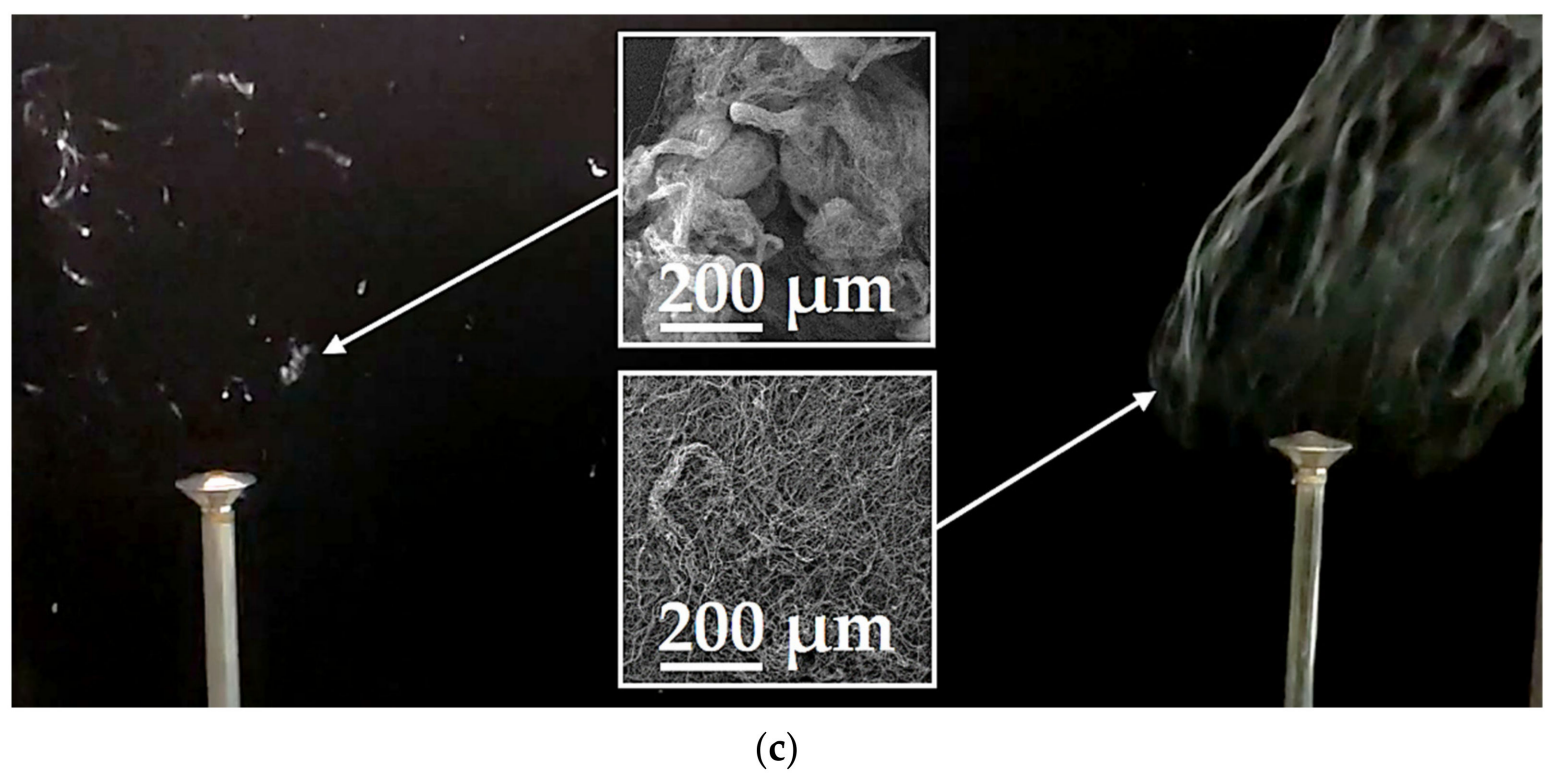
Scheme of the experimental setup (**a**) consisting of an electrode with a screw pump containing the polymeric solution, a high-voltage power source (TREK) with a signal generator (GEN-Owon AG 1022), a high-speed camera (i-SPEED 720), a DAQ module (NI USB-6216), and a computer (PC). An image of the screw pump electrode is shown on the right (**b**), consisting of a platform with a magnetic clutch (A), a polymer solution reservoir with a magnetic screw feeder (B), a steel overflow electrode (C), and a self-supporting nanofibrous product (D). The comparison of basic (**c**) **left** and enhanced solution (**c**) **right** and their nanofibrous products are also shown. Both electrodes were recorded at the same time, and both were powered using one power source.

The movement of the nanofibers was detected by an i-SPEED 720 high-speed camera system with a Nikon F-mount lens connection and a recording frequency of 10,000 Hz so as to obtain a maximum picture resolution of 1600 × 1200 px. The camera employed a CMOS sensor with dimensions of 27.972 × 20.736 mm. The high-intensity light source comprised an ILP-2 with a 100 W discharge lamp at a color temperature of 8200 K. This external unit provided illumination that was transmitted to the viewing area by a light guide cable and, subsequently, through the scope via the integral fiber bundle, to the viewing tip. The high-speed camera was placed in the vicinity (500 mm) of the top of the electrode, upon which the electrospinning took place (Figure 1).

### 2.5. Data Collection and Evaluation

**Figure 2:**
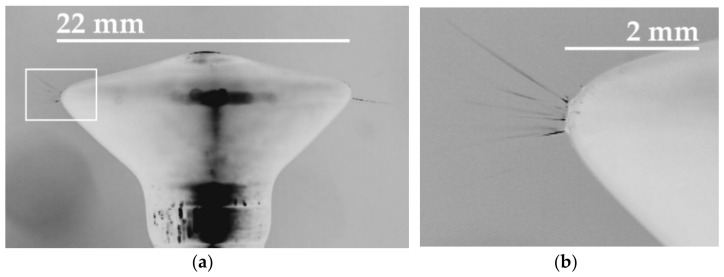
A disc-shaped steel overflow electrode (**a**) was used in the experiments. The white rectangle shows a detail of the edge (**b**) of the electrode on which the high-speed camera focused. The creation and subsequent collapse of the jets was observed and recorded only on the edge of the electrode tip due to the presence of the highest level of electrical intensity.

The required data were obtained using an NI USB-6216 multifunction DAQ device, synchronized with slow motion video images recorded on the high-speed camera. Because we connected the camera system sync and trigger connections to the DAQ hardware and used I-SPEED Suite 2.0 software to configure the data channels, the camera was automatically locked to the synchronization pulses sent by the data device. The prepared solutions were poured into the screw pump electrode [15], which was then connected to the high-voltage (HV) amplifier that served as a transformer for the low-voltage sinus or step function provided by the signal generator. The top of the spinning electrode was fully covered with the polymeric solution due to the overflowing mechanism of the pump (Figure 2a). The electrospinning process occurred on the edges of the top part of the electrode due to the presence of the highest electrical intensity (Figure 2b). I-SPEED Suite 2.0 software was used for the analysis of the recordings obtained. The color of the grayscale recordings was then inverted so as to enhance the visibility of the jets.

### 2.6. Morphology Analysis

The SEM pictures of the prepared nanofibers covered with a 10 nm layer of gold were obtained using a TESCAN Vega 3 SEM microscope. The diameters of the nanofibers were measured using ImageJ software. Each sample was measured 500 times followed by the creation of corresponding histograms.

## 3. Results

The experiment compared the reaction of basic and enhanced solutions with oxoacid to two types of signals: the sine wave and the step function. The reaction of the solutions is presented in graph form and as a series of close-up images of the electrospinning process (based on the position in the applied signal). The effective spinning area was characterized by an increased number of jets and their diameters in a certain part of the half-wave or step function signal.

### 3.1. AC Electrospinning Driven by the Sine Wave

**Figure 3:**
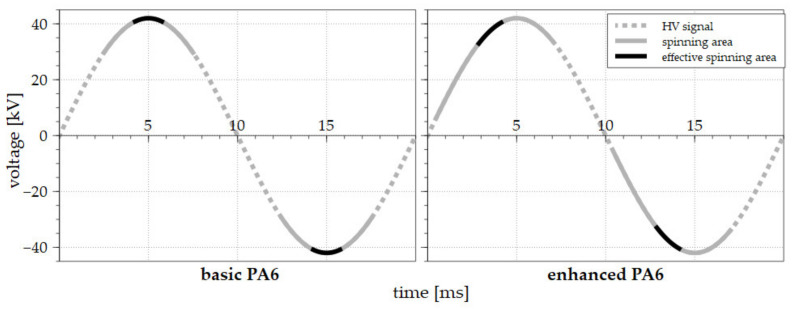
The AC electrospinning processes driven by the sinus wave signal for the basic PA 6 solution (**left**) and the enhanced solution (**right**). The voltage signals are represented by the dotted gray lines, the spinning areas are shown as a solid gray line, and the effective electrospinning areas are highlighted by solid black lines.

The basic PA 6 solution behaved as expected while exposed to AC high voltage driven by the sine wave. The solution destabilized and created the first Taylor cones and, subsequently, jets, after traversing around 30 kV (Figure 3 left). The maximum number of jets was observed in the amplitude area. The number of jets diminished following the exceeding of the amplitude. The collapse of the last Taylor cone was observed when the decreasing HV signal intersected a value of around 30 kV. The comparison of the positive and negative half-waves of the signal revealed that the average electrospinning time was approximately the same, i.e., both half waves electro-spun for around 50% of the signal time. It is important to mention here that despite the fact that the creation of Taylor cones and, subsequently, jets was observed during the spinning of the basic PA 6 solution, no fibrous layer was produced. While such an approach works well concerning DC electrospinning, electrospinning applying an AC power source led to the emission of fibrous flakes only [10].

**Figure 4:**
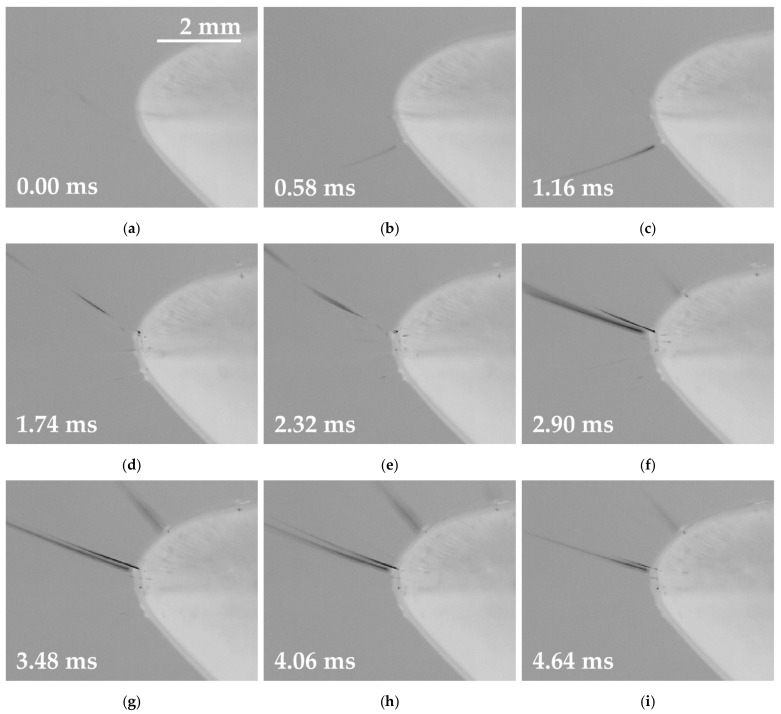

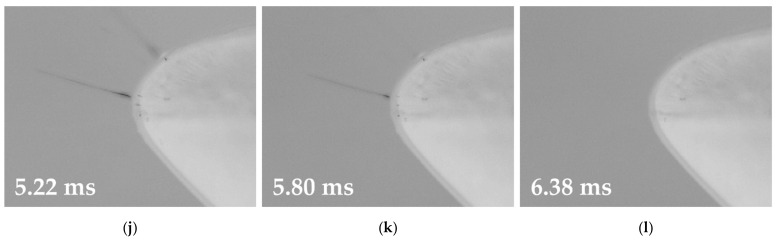
A frame sequence showing the accelerated electrospinning of the modified PA 6 solution driven by the sinus wave signal. The electrode with no instability (**a**) generated the first Taylor cone (**b**,**c**), followed by the creation of multiple cones (**d**,**e**). The efficient spinning area was characterized by a high number of thicker cones (**f**–**h**). The cones slowly diminished (**i**,**j**). The last jet (**k**) collapsed, thus leading to the initial state (**l**), which lasted for the rest of the half-wave (around 3.62 ms).

The next step involved the exposure of the two basic solutions supplemented with small doses of the oxoacids (0.20 mol/L H_2_SO_4_ or 0.37 mol/L CH_3_SO_3_H) to identical conditions and the evaluation of the results. This minor change in the composition of the solutions led to a major shift in the electrospinning offset. The graph in Figure 3 (right) reveals that the start of the electrospinning process occurred almost immediately after the signal crossed the zero-voltage value and lasted until the signal intersected the amplitude. A frame-by-frame detail of the spinning for the half-wave is shown in Figure 4. This process was similar with respect to both the positive and negative half-waves; moreover, the results were almost identical for the two additives. The highest number of jets was again observed in the middle of the spinning area, and the process lasted almost the same time as for the basic solution. In total, the enhancing of the basic solution led to the earlier beginning of the spinning process and an increase in the electrospinning time for both half-waves from 50% to approximately 60%.

The results suggest both a shift in the spinning area of the enhanced PA 6 solution and certain limitations in terms of the efficiency of the process regarding the maximum electrospinning time in any given half-wave. The earlier the offset, the sooner the self-termination of the process, even if the solution retains sufficient electrical energy for the continuation of the process. Such behavior suggests the depletion of the macromolecules that are arranged for electrospinning during any given signal half-wave.

### 3.2. AC Electrospinning Driven by the Step Function

**Figure 5:**
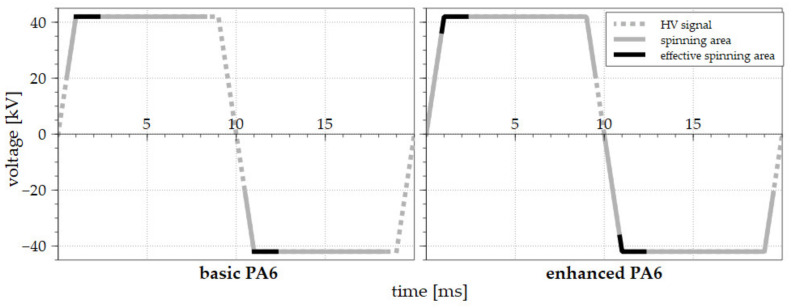
The AC electrospinning processes driven by the step change signal for the basic PA 6 solution (**left**) and the enhanced solution (**right**). The voltage signals are represented by the dotted gray lines, the spinning areas are shown as solid gray lines, and the effective electrospinning areas are highlighted by the solid black lines. The rising slope time was 1 millisecond.

The polymeric solution was subsequently exposed to a different type of high-voltage signal. The step function was chosen as a non-harmonic signal variant. However, due to the physical limitation of the high-voltage transformation, it proved impossible to obtain the ideal step function (Figure 5).

**Figure 6:**
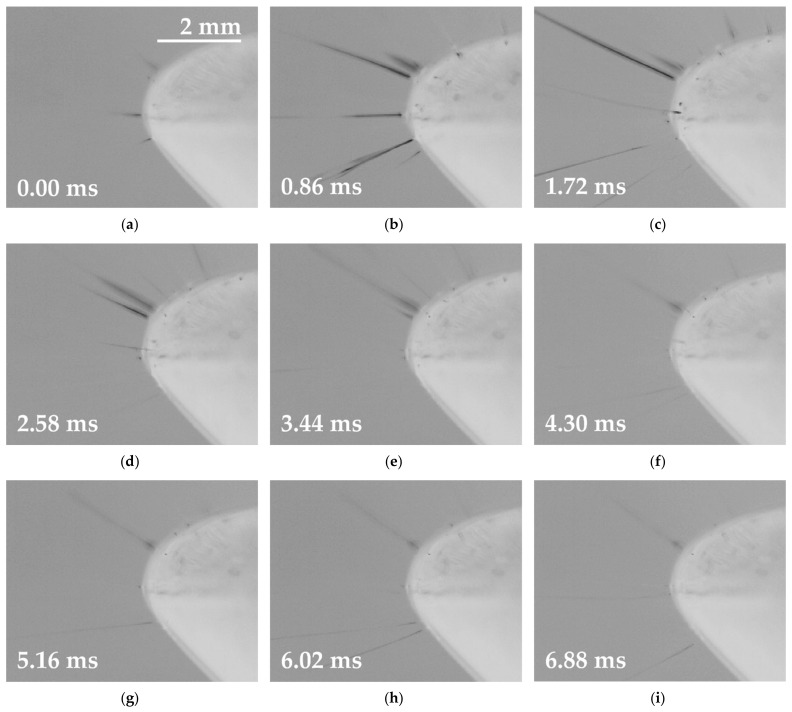

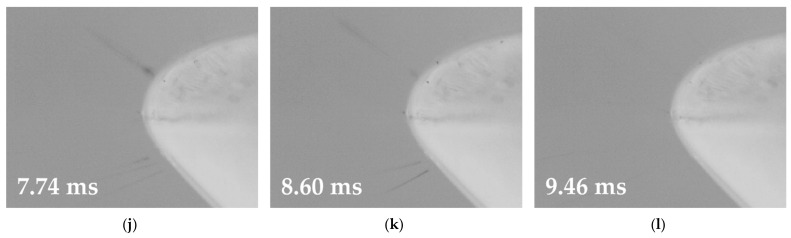
A frame sequence showing the accelerated electrospinning of the modified PA 6 solution driven by the step function. Upon traversing the zero line (**a**), the cones from the previous pulse were still visible; this was followed by the rapid offset of efficient electrospinning (**b**,**c**). A reduction in the efficiency of the process was visible (**d**,**e**), which led to a stable but less efficient process (**f**–**j**) for the rest of the pulse. The collapse of the weakening jets (**k**) led to an occasional state without the presence of any local instability (**l**), which lasted for the rest of the half-wave (around 0.54 ms).

The measurement of the step change function revealed two important phenomena, i.e., that the electrospinning offset occurred almost instantly, and that effective electrospinning was time-limited (Figure 5, right), which was also indicated during the previous sine wave experiment. The analysis of the step function signal (Figure 6) indicated that after reaching a value of 42 kV, effective electrospinning was visible for only approximately 1/10 of the period. During the rest of the ongoing signal, the solution managed to produce only a limited number of jets (a small fraction when compared to the effective spinning area). With respect to the use of the basic PA 6 solution, again, effective electrospinning was observed for only a short period of time (3/10 of the period) and immediately following the attainment of the maximum voltage. Only a limited number of cones was observed during the rest of the spinning time, thus indicating that a polymeric solution exposed to electric potential alone is not sufficient to ensure optimum electrospinning. This behavior is difficult to explain, especially in the case of a step function (Figure 6) that should, in theory, electro-spin fibers in similar quantity during the entire amplitude time. This is clearly not the case when examining the number of jets between Figure 6b–k. Further development of experimental methods that would allow for the examination of the composition of solution during the electrospinning in a high-voltage field could provide such an explanation.

An overview of all the experiments accompanied by the recorded data is presented in Table 1. All data in the table were recorded and measured 30 times, and average values with corresponding standard deviation are presented. Additives increased the time of the electrospinning per period independently of the signal shape used. The shortest spinning time was recorded using the basic solution and applying the sine wave signal (at around 50% of the of the period). Conversely, a general increase in the spinning time was recorded for the same electrode filled with the solutions containing the additives. The enhanced PA 6 solutions (with both H_2_SO_4_ or CH_3_SO_3_H) driven by the step function evinced the longest spinning times, i.e., in excess of 90% of both half waves. The examination of the experiments with regard to the dependency of the spinning time on the polarity of the signal revealed only minor differences.

The visual analysis of all the high-speed camera recordings (Figure 4 and Figure 6) highlighted the similarity in terms of the spinning behavior of the samples. The samples underwent four phases when exposed to the HV signals. Firstly, the solutions reacted via the creation of a small number of initial jets, while the second phase witnessed the highest number of visible jets (efficient spinning process). The third phase was characterized by weak electrospinning, followed by the collapse of the last jets (phase 4). These developments were observed for both types of signals, and both signal polarities. The dependency of productivity on the frequency of the signal is shown in Table 2. The productivity was determined as the weight of the fibrous layer produced in 60 min by a single electrode. It is assumed that a certain frequency limit exists for each polymer solution. After exceeding this frequency, no nanofibers of good quality can be obtained.

All the productivity measurements were taken after 24 h of the conditioning of the samples (25 °C, 50% relative humidity) so as to allow for the evaporation of the residual solvents that acted to increase the weight of the samples. All the measured samples were produced using the same type of solution and the same type of electrode (as described in the article).

**Figure 7:**
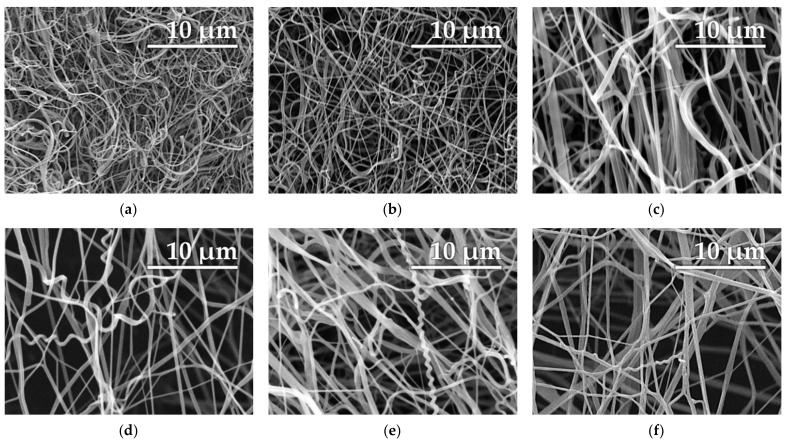
SEM images of the prepared PA 6 nanofibers: the basic PA 6 solution exposed to the sinus wave (**a**) and the step change (**b**), the PA 6 solution enhanced with sulfuric acid exposed to the sinus wave (**c**) and the step change (**d**), and the PA 6 solution enhanced with methane-sulfonic acid exposed to the sinus wave (**e**) and the step change (**f**). The images indicate a minimal change in the morphology compared to the two enhanced solutions.

**Figure 8:**
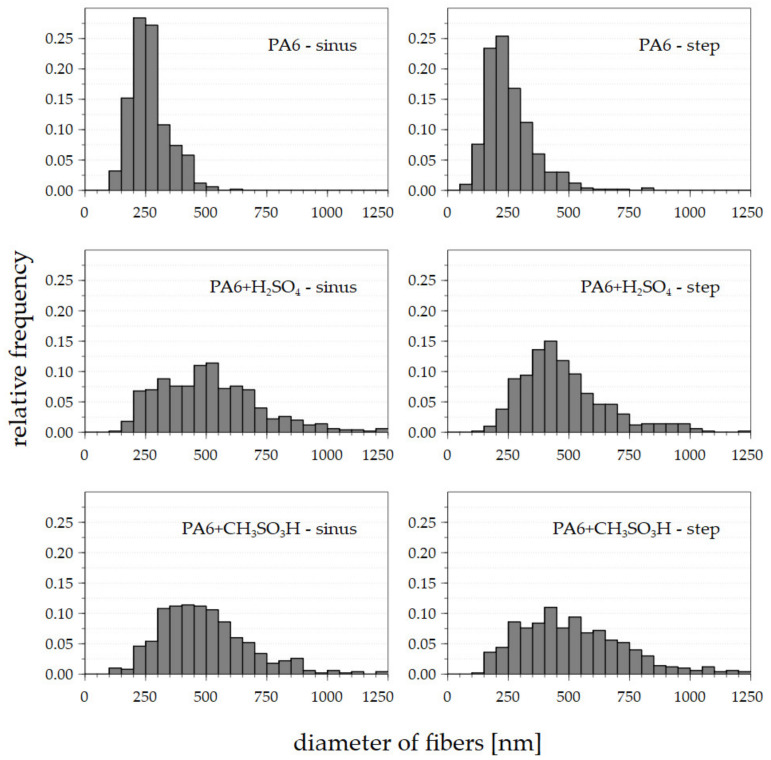
Histograms of the diameters of the nanofibers were created considering 500 values for each sample.

The modified solutions produced good quality nanofibrous materials (Figure 7), even though the diameters of the nanofibers (Figure 8) were greater than the diameters of the basic solution fibers [19]. It is important to note that it was impossible to effectively collect nanofibers from the basic solution due to the flaky character of the material. Such behavior renders large-scale production difficult and unfeasible at the industrial level.

The dominant peaks (Figure 8) of the fibers prepared from the basic solutions were between approximately 200 and 300 nm, and there were no major differences between the sinus wave and the step change. Conversely, the diameters of the enhanced solutions were greater with a wider range of diameters, thus rendering it difficult to select a dominant peak. Nevertheless, no major differences were detected between the diameters of the fibers according to the differing voltage functions or the additive. The graphs correspond to the SEM images shown previously.

The numerical description of histograms (Figure 8) is given in Table 3. The nature of AC electrospinning provides a wide area of fibrous diameters (standard deviation). The reason for this behavior is visible in Figure 4 and Figure 5, where the jets of thinner and thicker diameters are presented. This is also supported by the SEM images (Figure 7), where fibers with a wide range of diameters are visible.

## 4. Conclusions

The experimental results led to the conclusion that a minor difference in the composition of the polymeric solution led to a major change in the spinning behavior when exposed to an AC electric field. It was also confirmed that the shape (or frequency) of the signal played an important role in the optimization of the process and the productivity thereof. The high-speed camera recordings revealed that when exposed to an AC field, the PA 6 solution with the additive evinced an accelerated electrospinning offset. The basic PA 6 solution created jets for a shorter time than did the enhanced solution when exposed to the AC field. It is also worth mentioning that the basic solution was unable to produce a fibrous layer and created only small fibrous flakes that were technologically unprocessable. The additive-enhanced (oxoacids) solution variant was observed to spin over a longer time period and produced a solid bulky layer of nanofibrous material. It is assumed that each polymeric solution requires a certain frequency and shape of the signal for improved productivity of the nanofibers of sufficient quality. The further investigation of this phenomenon will be performed employing a wider range of solutions, frequencies, and signal shapes. The proper exploration of the spinning process is essential for the precise control of the electrospinning, thus improving the quality of complex materials for drug delivery systems, bioengineering composites, or chemical analysis.

## Figures and Tables

**Table 1:** Overview of the three PA 6 solutions and their reaction to the HV signal.

	PA 6		PA 6 + H_2_SO_4_ (0.2 mol/L)		PA 6 + CH_3_SO_3_H (0.37 mol/L)	
Sine function (polarity)	+	−	+	−	+	−
Spinning time (ms)	4.6	5.1	6.4	6.2	6.1	5.8
Standard deviation (ms)	1.2	0.5	0.3	0.4	0.5	0.7
Spinning time (%)	46	51	64	62	61	58
Step function (polarity)	+	−	+	−	+	−
Spinning time (ms)	8.2	7.6	9.4	9.6	9.6	9.3
Standard deviation (ms)	1.0	1.3	0.3	0.3	0.1	0.2
Spinning time (%)	82	76	94	96	96	93

**Table 2:** Productivity change of the PA 6 solution with H_2_SO_4_ when exposed to various step change signal frequencies.

Frequency (Hz)	50	100	150
Productivity sine wave (g/h)	1.51	2.86	4.46
Productivity step function (g/h)	2.73	4.51	6.15

**Table 3:** The average diameter of PA 6 nanofibers based on the solvent system and their standard deviation.

Solution	Basic		Basic + H_2_SO_4_		Basic + CH_3_SO_3_H	
Signal	Sinus	Step	Sinus	Step	Sinus	Step
Average diameter (nm)	265	257	517	480	501	521
Standard deviation (nm)	79	104	215	185	205	225

## Data Availability

The data presented in this study are available on request from the corresponding author.
